# Individual Effector/Regulator T Cell Ratios Impact Bone Regeneration

**DOI:** 10.3389/fimmu.2019.01954

**Published:** 2019-08-16

**Authors:** Claudia Schlundt, Simon Reinke, Sven Geissler, Christian H. Bucher, Carolin Giannini, Sven Märdian, Michael Dahne, Christian Kleber, Björn Samans, Udo Baron, Georg N. Duda, Hans-Dieter Volk, Katharina Schmidt-Bleek

**Affiliations:** ^1^Julius Wolff Institut and Center for Musculoskeletal Surgery, Charité – Universitätsmedizin Berlin, Berlin, Germany; ^2^Berlin-Brandenburg Center for Regenerative Therapies, Charité – Universitätsmedizin Berlin, Berlin, Germany; ^3^Institute of Medical Immunology, Charité – Universitätsmedizin Berlin, Berlin, Germany; ^4^Center for Musculoskeletal Surgery, Charité – Universitätsmedizin Berlin, Berlin, Germany; ^5^University Center of Orthopaedics and Traumatology, University Medicine Carl Gustav Carus Dresden, Dresden, Germany; ^6^Epiontis GmbH, Precision for Medicine Group, Berlin, Germany; ^7^Berlin Center for Advanced Therapies (BeCAT), Charité – Universitätsmedizin Berlin, Berlin, Germany; ^8^Berlin Institute of Health Center for Regenerative Therapies, Berlin, Germany

**Keywords:** bone healing, regeneration, effector T cell, regulatory T (Treg) cell, mouse model

## Abstract

There is increasing evidence that T lymphocytes play a key role in controlling endogenous regeneration. Regeneration appears to be impaired in case of local accumulation of CD8+ effector T cells (T_EFF_), impairing endogenous regeneration by increasing a primary “useful” inflammation toward a damaging level. Thus, rescuing regeneration by regulating the heightened pro-inflammatory reaction employing regulatory CD4+ T (T_Reg_) cells could represent an immunomodulatory option to enhance healing. Hypothesis was that CD4+ T_Reg_ might counteract undesired effects of CD8+ T_EFF_. Using adoptive T_Reg_ transfer, bone healing was consistently improved in mice possessing an inexperienced immune system with low amounts of CD8+ T_EFF_. In contrast, mice with an experienced immune system (high amounts of CD8+ T_EFF_) showed heterogeneous bone repair with regeneration being dependent upon the individual T_EFF_/T_Reg_ ratio. Thus, the healing outcome can only be improved by an adoptive T_Reg_ therapy, if an unfavorable T_EFF_/T_Reg_ ratio can be reshaped; if the individual CD8+ T_EFF_ percentage, which is dependent on the individual immune experience can be changed toward a favorable ratio by the T_Reg_ transfer. Remarkably, also in patients with impaired fracture healing the T_EFF_/T_Reg_ ratio was higher compared to uneventful healers, validating our finding in the mouse osteotomy model. Our data demonstrate for the first time the key-role of a balanced T_EFF_/T_Reg_ response following injury needed to reach successful regeneration using bone as a model system. Considering this strategy, novel opportunities for immunotherapy in patients, which are at risk for impaired healing by targeting T_EFF_ cells and supporting T_Reg_ cells to enhance healing are possible.

## Introduction

The most promising healing scenario for a damaged tissue would be self-repair, thus to regenerate. Bone is able to heal without scar formation and therefore has a high regenerative capacity (1). Therefore, the healing process after bone injury could serve as a blue print for understanding underlying mechanisms guiding successful tissue regeneration. The majority of bone fractures heal by endochondral ossification, which is usually divided into five distinct but overlapping stages. Bone healing starts with an initial hematoma formation and inflammation (further divided into pro- [1] and anti-inflammation [2]), subsequent revascularization of the fracture area and cartilage formation (soft callus formation [3]) occurs. Chondrocyte hypertrophy and matrix calcification proceed its replacement by newly formed woven bone (hard callus formation [4]). The final step of the healing cascade is the remodeling [5] of the new formed bone in dependence on the mechanical stimuli during loadbearing. In this generally successful healing of bone, immunological cells play a core role and impact the cascades of regeneration across all of these stages.

Despite its significant endogenous regeneration potential, in a meaningful proportion of humans (10–15%) fracture healing does not succeed and results in delayed union or persistent non-union (2, 3). This still represents a considerable health care problem, specifically in the aging industrial societies (4). After fracture, the initial pro-inflammatory reaction dominated by M1 macrophages and Th1 T cells and their chemokine and cytokine release pattern initiates the infiltration of cells needed for a successful repair process (5, 6).The switch from an initial pro-inflammatory to a subsequent anti-inflammatory state has been proven to be the key to any successful healing (7). There is growing evidence that the adaptive immunity, in particular, T cells, contributes to endogenous regeneration even in the absence of infections (8, 9) by modulating the local cytokine milieu in the fracture gap (8, 10–15) and thus during this early inflammatory phase. Using samples from fracture patients, it was recently shown that a higher amount of CD8+ effector T cells (T_EFF_) is associated with a prolonged pro-inflammatory reaction and impaired fracture repair (16, 17). Accordingly, systemic depletion of CD8+ T cells in a mouse osteotomy model enhanced endogenous fracture regeneration, whereas the adoptive transfer of CD8+ T cells impaired the regeneration process. Therefore, high CD8+ T effector cell percentages are critical for an uneventful healing.

Important counterparts of an unfavorable pro-inflammatory immune response are CD4+ regulatory T cells (T_Reg_). CD4+ T_Reg_ are a highly specialized cell population with immunomodulatory functions and are characterized by the expression of the surface marker CD25 and the transcription factor Forkhead-Box-Protein P3 (FOXP3) (7, 18–21). These cells are essential for the maintenance of the immunological self-tolerance, but also to prevent overwhelming inflammation in response to pathogens and in response to tissue injury. Several studies show that CD4+ T_Reg_ might control osteoclast activity and as result osteoarthritis, and enhance bone formation as well as inhibit bone loss under physiological and pathological conditions (19–21). CD4+ T_Reg_ execute immunomodulatory functions by multiple mechanisms, such as cell-cell contact dependent and independent ones. Their direct impact on the ATP metabolism via the surface molecules CD39 and CD73 results in the generation of strong immunomodulatory ATP derivatives, such as adenosine, and might be of particular interest for their immune-regulatory potency in inflamed tissues. Adenosine inhibits cytokine secretion by and proliferation of activated T_EFF_ cells as well as the switch of tissue resident M2 macrophages to pro-inflammatory M1 macrophages (22–24).

With the growing evidences that activated T_EFF_ negatively impact regenerative processes, their counterparts, the CD4+ T_Reg_ might have a positive regulatory impact on endogenous regeneration. In fact, there are a couple of studies showing their pro-regenerative potency e.g., after myocardial infarction or in regeneration from myocarditis (22).

We hypothesize that CD4+ T_Reg_ possess the potential to reshape and further to rebalance an initial unfavorable and “anti-regenerative” immune status (e.g., created by an increased CD8+ T_EFF_ level) into a pro-regenerative state promoting successful bone healing after fracture.

The role of the adaptive immunity in regeneration was overlooked for a long time, mainly because of using mouse models with a naïve T cell phenotype (23). Conventionally, specific pathogen free (SPF) housing is applied to have more standardized conditions. However, this housing is silencing the adaptive immunity, and even >1 year old SPF mice express an almost naive immune system lacking T_EFF_ cells as seen in newborns or babies but not comparable to adult human beings who are permanently exposed to environmental challenges (24). We further hypothesize that immune experienced mice kept under non-SPF, and thus pathogen exposed, conditions are the clinically more relevant model for studying the role of the T_EFF_/T_Reg_ balance in bone regeneration.

Within this study the interplay of CD8+ T_EFF_ and CD4+ T_Reg_ was analyzed using immunologically naïve SPF housed mice (low CD8+ T_EFF_) and non-SPF housed animals (high percentage of T_EFF_) to mimic the impact of immune experience. Finding were compared to human fracture samples from patients with a known undisturbed healing and a diagnosed delayed healing outcome. Findings demonstrate the significant impact of a balanced T_EFF_/T_Reg_ ratio on bone regeneration and at the same time opens novel avenues for personalized immunomodulatory therapies.

## Materials and Methods

### Study Design

To evaluate the potential of CD4+ T_Reg_ to reshape an initial unfavorable and “anti-regenerative” immune state into a “pro-regenerative” one, we went into our well-established mouse osteotomy model system. Freshly isolated murine CD4+ T_Reg_ were adoptively transferred into mice prior to osteotomy. The healing outcome was investigated 21 d post-surgery. To have a clinically more relevant mouse model, we included for the immunomodulatory approach an osteotomy model using mice possessing a more experienced adaptive immune system (*n* = 20; control: *n* = 6, with adoptive CD4+ T_Reg_ transfer: *n* = 15 [non-SPF housing]) compared to the classically used naïve mice (*n* = 12; control: *n* = 6, with adoptive CD4+ T_Reg_ transfer: *n* = 6 [SPF housing]). One mouse was excluded from the study due to a non-evaluable healing outcome as a consequence of a failed fixation. In the non-SPF housed mice, the CD8+ T_EFF_ to CD4+ T_Reg_ ratio was evaluated by flow cytometry pre- as well as post-osteotomy to investigate the interplay of the cell ratio and the healing outcome (Figure S1: Study design of the mouse experiment).

We included data from human fracture patients to confirm the murine findings in a patient setting. Thirty-five patients (aged 19–77 years, 19 male, and 16 female) were included in this study. Due to the assessment of immunological parameters, patients with human immunodeficiency virus infection, hepatitis infection, ongoing, or past (within 5 years) malign diseases/treatments were excluded from the study. To ensure a similar post-surgery physiotherapeutically mobilization, patients with polytrauma or several fractures (>2 fractures) were also excluded from the study (see Table 1, Tables S1, S2). Based on the radiological data, fracture patients were divided into normal (*n* = 23) and impaired (*n* = 12) healing fractures. To analyze the interplay of the CD8+ T_EFF_ to CD4+ T_Reg_ ratio and the healing outcome, peripheral blood samples were taken prior to surgery, and the T_EFF_/T_Reg_ was analyzed by flow cytometry. In addition, the cell ratio was also investigated in fracture hematoma samples, taken during the surgery.

**Table 1:** Characterization of the patients.

**Parameter**	**Impaired healing patients (*n* = 12)**	**Normal healing patients (*n* = 23)**	**Significance**
Age (y)	54.58 ± 12.7	52.17 ± 13.3	0.6
Sex (male/female)	7/5	12/11	0.7
Height (m)	1.71 ± 0.09	1.74 ± 0.09	0.3
Weight (kg)	79.63 ± 18.52	76.43 ± 15.79	0.6
BMI	27.3 ± 5.9	25.2 ± 4.2	0.2

### Characteristics of the Animal Model Used for Immune Intervention Approaches

Female C57BL/6 mice (Charles River Laboratories, Wilmington, USA) of an age of 12 weeks were used for the animal experiments. Mice were kept in small groups under specific pathogen free (SPF) or non-SPF (area in the animal facility without an additional barrier and filtered air supply for the mice cages) housing conditions. A controlled temperature (20 ± 2°C) and a 12 h light/dark circle were present. Food and water were available *ad libitum*. All animal experiments were approved by the local legal representatives (Landesamt für Gesundheit und Soziales Berlin: G0008/12; T0119/14; T0249/11) and done accordingly to the guidelines of the Animal Welfare Act, the National Institutes of Health Guide for Care and Use of Laboratory Animals, and the National Animal Welfare and ARRIVE Guidelines. Health monitoring followed the FELASA guidelines for both housing conditions—no health risks were monitored for those animals housed under non-SPF conditions.

CD4+ T_Reg_ were enriched by cell sorting (see below) and adoptively transferred (5–8 × 10^5^ cells) via the tail vein prior to surgical intervention. Mice were randomly allocated to the treatment groups.

### Surgical Approach and Performing the Mouse Osteotomy

A non-critical sized osteotomy was set in the left femur of the mice as described before (6). In short, mice were anesthetized by inhalation of isoflurane and received a subcutaneous injection of the analgesic Buprenorphine (0.03 mg/kg) and of the antibiotic Clindamycin (45 mg/kg). After shaving the surgery field and disinfection, a longitudinal cut of the skin was performed. The femur was bluntly exposed and the external fixator, consisting of four pins and a bar (RISystem, Davos, Switzerland), was mounted on the lateral side of the femur. An osteotomy of 0.7 mm was created between the two middle pins using a wire saw. The skin was sutured, and the mice were brought back into their cages. As post-operative analgesia, tramadol hydrochloride (25 mg/l) was added to the drinking water for 3 days post-surgery.

### Isolation of Murine Regulatory T Cells for Immunotherapy

Murine CD4+ regulatory T cells (T_Reg_) were isolated by magnetic activated cell sorting (MACS) from pooled cells derived from the spleen and the lymph nodes: inguinal nodes, axillary nodes, brachial nodes, and mesenteric nodes. A single cell suspension was prepared and erythrocytes were lysed. The isolation procedure was carried out with the CD4+ T_Reg_ isolation kit (Miltenyi Biotec GmbH, Bergisch Gladbach, Germany), according to the manufactures instructions. Briefly, the cell suspension was incubated with a biotin-labeled antibody mix to stain all non-CD4+ T cells. Afterwards, α-biotin magnetic beads, as well as a PE-labeled α-CD25 antibody were added to the cells. After incubation, cells were washed (8 min, 1,500 rpm, 4°C), resuspended in MACS buffer (1x PBS + 0.5% BSA + 2 mM EDTA) and transferred to an LD column (Miltenyi Biotec GmbH, Bergisch Gladbach, Germany), which was placed in a magnetic field. The flow through was collected, washed, resuspended in MACS buffer and incubated with an α-PE antibody. Cells were washed, resuspended in MACS buffer and transferred to an MS column (Miltenyi Biotec GmbH, Bergisch Gladbach, Germany), which was placed in a magnetic field. CD4+ T_Reg_, which were kept in the magnetic field in the column, were flushed out, washed and counted. Subsequently, CD4+ T_Reg_ were isolated with a purity of ≥85%.

### Regulatory T Cell Suppression Assay

The suppressive capacity of freshly isolated CD4+ T_Reg_ was tested in a co-culture setup with CD4+CD25- responder T cells (T_Responder_). T_Responder_ were labeled with a cell proliferation dye (Cell Proliferation Dye (CPD) eF450, eBioscience, San Diego, USA) following the protocol. In short, MACS isolated T_Responder_ were washed (8 min, 1,500 rpm, RT) twice with PBS and resuspended in a solution consisting of pre-warmed PBS and a 10 mM CPD eF450 solution to a final concentration of 10 × 10^6^ cells/ ml. Cells were incubated for 20 min at RT in the dark and the staining was stopped by the addition of ice cold cell culture media [RPMI 1640 (Biochrom AG, Berlin, Germany) + 10% FBS superior (Biochrom AG, Berlin, Germany) + 1% penicillin/streptomycin (Sigma-Aldrich Chemie GmbH, München, Germany), and 50 μM β-Mercaptoethanol (Sigma-Aldrich Chemie GmbH, München, Germany)]. Cells were incubated for 5 min on ice, washed three times with pre-warmed cell culture media and counted. For the suppression assay, 0.75 × 10^5^ labeled T_Responder_ were co-cultured in cell culture media with (1) 1.5 × 10^5^ or (2) 0.15 × 10^5^ CD4+ T_Reg_ in a 96 well plate. T_Responder_ were also cultured alone. In order to stimulate the proliferation of T_Responder_, α-CD3 and α-CD28 (both eBioscience, San Diego, USA) were plate-bound (each 5 μg/ml) to the wells before adding the co-culture setups. After 2–3 days, cells were harvested, and the proliferation of the T_Responder_ was analyzed by flow cytometry. The suppression of the CD4+ T_Reg_ was calculated based on the proliferation of the T_Responder_ which were cultured alone. Their proliferation was set to 100, and the proliferation of the co-cultured T_Responder_ was calculated relative to it. Finally, the percentage of suppression was defined as 100 minus the (relative) proliferation.

### Flow Cytometry Analysis of Murine Immune Cells in Tissue Samples

The sample preparation for flow cytometry was done as follows: from the spleen, the bone marrow, and the lymph nodes, single cell suspensions were prepared. The spleen was cut into small pieces and carefully pushed through a cell strainer. For the bone marrow, the ends of the bones were cut and the marrow was flushed out with a syringe. Afterwards, the bone marrow cells were also carefully pushed through a cell strainer. Lymph nodes were directly pushed through a cell strainer. All single cell suspensions were washed in PBS, and the erythrocytes were lysed. Blood samples were centrifuged (10 min, 2,000 rpm, RT), the supernatant was removed, and erythrocytes were lysed. Cells were washed in PBS (10 min, 1,500 rpm, 4°C) and stained. Lysed and washed cells were resuspended in PBS and incubated with a LIVE/DEAD staining kit (Thermo Fisher Scientific, Waltman, USA) for 30 min on ice. For the evaluation of the adoptive CD4+ T_Reg_ transfer (1 and 21 days post-osteotomy) and the amount of CD4+ T_Reg_ in the harvested tissues after 21 days of healing, cells were washed, resuspended in PBS/BSA (1x PBS + 1% BSA and 0.1% sodium azide) and stained with the following antibody mix for 20 min on ice: α-CD3 PerCP (BioLegend, San Diego, USA), α-CD4 AF700 (eBioscience, San Diego, USA) and α-CD25 APC (BioLegend, San Diego, USA). For the evaluation of the CD4+ and CD8+ effector T cell subset, cells were washed, resuspended in PBS/BSA and stained with the following antibody mix for 20 min on ice: α-CD3 PerCP (BioLegend, San Diego, USA), α-CD4 AF700 (eBioscience, San Diego, USA), α-CD8 eF450 (eBioscience, San Diego, USA), α-CD44 PE-Cy7 (Becton Dickinson Bioscience, Heidelberg, Germany), α-CD62L APC (BioLegend, San Diego, USA) and α-CD25 APC (BioLegend, San Diego, USA). Cells were washed, resuspended, and either fixed for 20 min at RT with a 2% formaldehyde solution (evaluation of adoptive CD4+ T_Reg_ transfer) or permeabilized with a fixation/permeabilization buffer (eBioscience, San Diego, USA) for 1 h on ice (evaluation of the amount of CD4+ T_Reg_ after 21 days). Cells were washed twice with permeabilization buffer (eBioscience, San Diego, USA), resuspended and incubated for 30 min on ice with α-Foxp3 FITC (eBioscience, San Diego, USA). After fixation and permeabilization, cells were washed, resuspended and analyzed with the LSR II flow cytometer (Becton Dickinson Bioscience, Heidelberg, Germany) (for the gating strategy please refer to Figure S2).

### Micro-Computed Tomography of Osteotomized Mouse Bones

To evaluate the formation of newly formed bone, μCT analyses were performed with the osteotomized bones 21 days post-surgery. The harvested bones were scanned in a μCT Viva 40 (SCANCO Medical AG, Brüttisellen, Switzerland). The volume of interest (VOI) included 190 slices around the former osteotomy gap. The following parameters were used for the scan: 10.5 μm voxel size, 55 keVp peak voltage energy and an applied current of 145 μA. In order to be able to distinguish between non-mineralized and mineralized bone, a gray value threshold was used (25). A defined threshold of 396.9 mg hydroxyapatite per cm^2^ identified mineralized bone.

### Patients and Study Protocol

The study was performed in compliance with the International Conference on Harmonization Guidelines for Good Clinical Practice and the Declaration of Helsinki. All patients participated with a written informed consent, and the study was approved by the Institutional Review Board (IRB) of the Charité – Universitätsmedizin Berlin (IRB approval EA2/096/11). All patients received a complete study follow up rate including all clinical investigation time points and radiological assessments. Moreover, all patients were matched according to age, sex, fracture type and initial surgical treatment strategy. The demographic and clinical characteristics of the included patients are shown in Table 1, Tables S1, S2. To monitor the fracture healing process and in consensus with established clinical examination time points, the patients were investigated pre-surgery and after 4–6, 12, 17–19, 24, and 52 weeks. Regarding the fracture treatment, the only significant difference between the healing groups was the number of surgical interventions required (*p* = 0.003; Table S2).

### Healing Classification and Data Collection

*Time-dependent criteria*: A fracture was categorized as impaired healing when it was not completely healed after 17–19 post-operative weeks based on the callus formation. *Radiological criteria:* Different criteria were applied to identify an impaired healing fracture by radiological analyses: (A) incomplete fracture healing or the absence of visible bone consolidation on a simple X-ray after 17–19 post-operative weeks; (B) the presence of a resorption zone or incomplete callus formation; (C) incomplete bridging, which means one to three cortices bridged; or (D) no bridging, which means no cortex is bridged.

Every patient underwent consecutive x-ray analyses to assess the stability of the implant and to observe the fracture gap throughout the study time. Appraisal of x-rays was performed by three independent, blinded specialists (two orthopedic surgeons and one radiologist), to ensure the healing outcome and the classification of patients as shown in Table 1. To fulfill the definition of impaired healing, patients had to meet one or more of the time dependent- and/or radiological criteria as stated above (26–29).

### Flow Cytometry Analysis of Human Blood Samples to Characterize Immune Status in Fracture Patients

Blood samples were taken before the surgery after 15 min rest in a supine position. All blood samples were immediately moved into a dark, air-conditioned room, and sent to the laboratory within 2 h. Additionally, plasma and serum samples were collected in aliquots and frozen at −80°C. Full blood count and standard clinical variables (erythrocytes, hemoglobin, hematocrit, thrombocytes, creatinine, sodium, potassium, urea, chloride, GPT, GOT, gamma-GT, TSH, CRP) were measured immediately in plasma and serum samples according to the laboratory standard operating procedures.

To evaluate the adaptive immunity of the patients, we applied our recently developed and extensively validated pre-cocktailed dried DuraClone T cell panels (Beckman Coulter), including the “T cell panel” CD45RA, CCR7, CD28, PD1, CD27, CD4, CD8, CD3, CD57, CD45, and “regulatory T cell panel” (Beckman Coulter), including CD45RA, CD25, CD127, CD39, CD4, FOXP3, CD3, CD45 were used. Flow cytometry analysis was performed using the BC NAVIOS 10/3 flow cytometer and data were analyzed using BC Kaluza analysis software (Beckman Coulter).

### Human Genomic DNA (gDNA) Purification for Epigenetic Analysis

Frozen human fracture hematoma samples (*n* = 8) were thawed in a 37°C water bath and further kept on ice. To dissolve the hematoma samples, Clotspin® Baskets from Qiagen were used according the manufacturing protocol: Purification of archive-quality gDNA from clotted whole blood using Clotspin® Baskets and the Gentra® Puregene® blood kit provided by Qiagen. All protocol steps were followed according to the manufacturer's instructions. Steps included the usage of isopropanol and Glycogen Solution (20 mg/ml from Qiagen) and washing of gDNA pellets with 70% ethanol. gDNA was further air dried at room temperature until no remaining fluids remained. gDNA was incubated with 500 μl DNA hydration solution provided within the kit (www.qiagen.com/literature/handbooks/default.aspx). gDNA was incubated at 65°C until it was dissolved.

gDNA quality and quantity were confirmed using the NanoDrop-ND-1000 system (PEQLAB GmbH). Samples were stored at −80°C until further experiments.

### Epigenetic qPCR Analysis (Patients)

Purified genomic DNA from the fracture hematoma (*n* = 8) was converted using the EpiTect Fast Bisulfite Conversion Kit (Qiagen) following the manufacturer's instructions. CD3+, T_Reg_ -specific and CD8B+ T cell-specific epigenetic qPCR analyses were performed as previously described (30–32).

### Statistics

For the statistical evaluation of the data, the program SPSS (Version 22; IBM Deutschland GmbH, Ehningen, Germany) was used. Unless otherwise stated, all data were represented as means ± SD.

#### Human Data

The Levene test was used to assess the homogeneity of variances of the data for the indicated groups and the data were then analyzed using the unpaired Student's *t*-test (two groups).

#### Mouse Data

Due to the small sample sizes, a normal distribution of the data was excluded. Therefore, the Mann-Whitney *U*-test was used as statistical test. If more than two samples were compared, the significance value p was corrected by using the Bonferroni correction. Data were seen as statistically significant if *p* ≤ 0.05 or if *p* ≤ 0.05/n (*n* = number of compared samples) (Bonferroni corrected data). Data are presented as scatter or boxplot graphs. Usage of the Bonferroni correction is indicated in the respective figures.

### Data and Materials Availability

All data needed to evaluate the conclusions in the paper are presented within the paper and/or in the Supplementary Materials. Additional data related to this paper may be requested from the corresponding author.

## Results

In a small cohort of patients, we previously showed that CD57+ CD8+ T_EMRA_ cells were markedly elevated in peripheral blood of patients suffering from an impaired bone healing (17). T cell receptor stimulation or IL-12 (bystander activation) can trigger T_EMRA_ cells to produce inflammatory cytokines, like IFNγ and TNFα, without the need of antigen-presenting cells (independent on co-stimulatory signals via CD28 or CD40L), making those ideal candidates for enhancing intra-tissue inflammation if they accumulate after injury. It is well-established that T_Reg_ play a crucial role in controlling immune response at multiple levels to prevent undesired immune reactivity, such as auto/allo-immunity and infection-related pathogenesis (33). There is increasing evidence that they are also pivotal in controlling regenerative processes (34). Therefore, we wondered whether adoptive transfer of CD4+ T_Reg_ might be a relevant option to enhance fracture healing. To address this we applied a well-defined mouse osteotomy model (35) and adoptively transferred CD4+ T_Reg_.

### Application of CD4+ Regulatory T Cells as Potential Agent to Improve Bone Fracture Healing in SPF Mice

**Figure 1:**
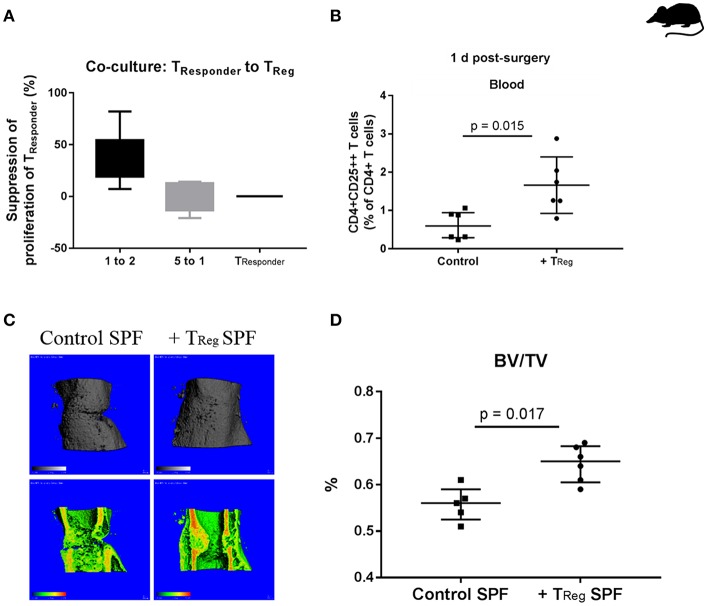
Enhancing bone healing by adoptive transfer of murine CD4+ T_Reg_ in SPF mice. **(A)** Demonstration of the functionality of isolated murine CD4+ T_Reg_ in a suppression assay, illustrated is the suppression of the proliferation of CD4+CD25- T_Responder_ cells by using freshly isolated CD4+ T_Reg_ (*n* = 6). **(B)** Status of CD4+ T_Reg_ in the peripheral blood of the experimental mice 1 d after adoptive transfer, control (*n* = 6) vs. CD4+ T_Reg_ enriched mice (*n* = 6). **(C,D)** Improved bone healing by adoptive CD4+ T_Reg_ transfer as demonstrated by μCT evaluation after 21 d, control (**C**, left, control SPF) vs. T_Reg_ enriched mice (**C**, right, +T_Reg_ SPF). Quantification of the μCT data revealed a significantly higher BV/TV in the mice that received a CD4+ T_Reg_ transfer (+T_Reg_) (*n* = 6) compared to the control SPF mice (*n* = 5) **(D)**. BV, bone volume; TV, total callus volume; BV/TV, ratio of BV to TV; Mann-Whitney *U*-test.

Prior to adoptively transferring the CD4+ T_Reg_, we tested the functionality of *ex vivo* enriched murine CD4+ CD25++ Foxp3+ T_Reg_. Using an established CD4+ T_Reg_ suppression assay in which freshly isolated CD4+ T_Reg_ (purity ≥85%, Figure S3) and stimulated T_Responder_ (non-T_Reg_) cells were co-cultured, we confirmed their suppressive properties (Figure 1A).

In order to confirm the successful transfer of the CD4+ T_Reg_ their fate was analyzed in the recipient mice. After infusion of CD4+ T_Reg_ into the tail vein of C57BL/6 mice prior to osteotomy, a blood sample for counting circulating CD4+ T_Reg_ was taken 1 day (1 d) after surgery. A significantly higher percentage of CD4+CD25++ T cells (representing CD4+ T_Reg_) was found in animals that received an adoptive transfer in comparison to control mice (Figure 1B) illustrating the efficacy of adoptive transfer of T_Reg_ (*p* = 0.015).

After proving the feasibility of isolation, transfer and functionality of adoptively transferred CD4+ T_Reg_, we tested our hypothesis that these cells had a positive impact on bone healing.

Initially we used SPF mice (12 weeks), where skeletal growth was nearly completed and peak bone mass has almost been reached (36). Further, an osteotomy model was used that did not results in complete healing within 21 days and thus allows to detect improvements in healing in comparison to control mice. As expected, all control mice expressed a healing with a partly visible osteotomy gap, as documented by μCT data (Figure 1C). The experiment confirmed our hypothesis showing improved bone healing at 21 d as indicated by a significantly increased ratio of bone volume to total callus volume (BV/TV) in the osteotomized femura of mice that received an adoptive transfer of CD4+ T_Reg_ (Figure 1D; *p* = 0.017).

In summary, the elevation of the CD4+ T_Reg_ in recipient mice showed a positive effect on bone healing. However, these mice were kept under the usual SPF housing conditions resulting in a “naïve” T cell system. In contrast, human beings are permanently exposed to environmental antigens shifting their T cell composition to higher T_EFF_ cell counts in an age-dependent manner (32). Therefore, alternative murine osteotomy models are required that better reflect the situation in human patients.

### Establishment of a Clinically More Relevant Murine Model Applying Non-SPF Housing to Generate an “Experienced” T Cell Immunity Reflected by Enhanced T_EFF_ Cell Counts

Recently, we established a new model of non-SPF housing conditions inducing a fast switch to a more “experienced” T cell immunity with enhanced levels of T_EFF_ cells (24, 37). Briefly, mice were transferred toward a non-barrier housing exposing them to environmental pathogens. Thus, the environmental microbial exposure was raised in comparison to the SPF housed mice and the adaptive immune system was challenged. TNAIVE/TMEMORY/T_EFF_ counting in a blood sample before osteotomy allows individual proof of “immune aging,” of immune experience. It is worth mentioning that after 5 years of experience with this model, no clinical abnormality was reported in these mice nor has any serological disease status been recorded in this cohort outside of an SPF barrier.

After a minimal exposure of 4 weeks, the immune cell composition in these non-SPF housed animals was analyzed and compared to that of SPF housed animals. Although there is a significant individual variation, mice developed a higher level of T_EFF_ (both CD4+ and CD8+) in immune tissues, e.g., spleen. Thus, the exposure led to an altered CD8+ T_EFF_/CD4+ T_Reg_ ratio in non-SPF mice compared to SPF mice (spleen: *p* = 0.008; Figure S4).

In the next step, we asked whether an adoptive transfer of CD4+ T_Reg_ could enhance bone healing in these mice with a more experienced immune system and a consecutively higher percentage of CD8+ T_EFF_.

### Adoptive Transfer of CD4+ T_Reg_ in Mice With an Experienced Adaptive Immunity Results in Heterogeneous Bone Healing Outcome

**Figure 2:**
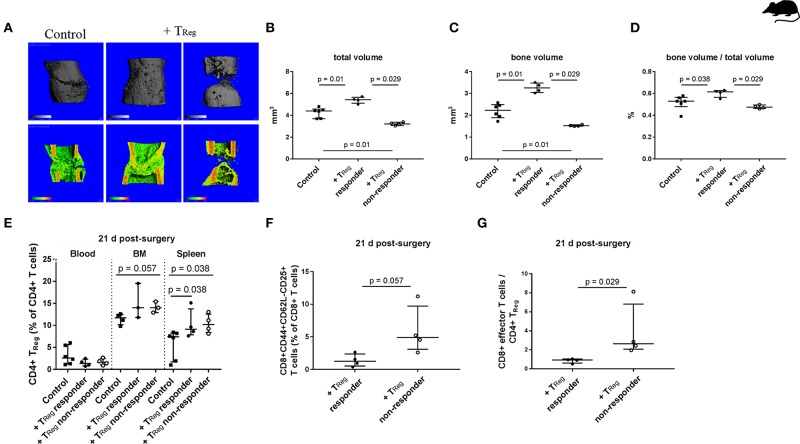
Heterogeneous impact of adoptive transfer of CD4+ T_Reg_ on bone healing outcome in mice kept under non-SPF housing conditions. **(A–D)** μCT evaluation of the healing outcome after adoptive CD4+ T_Reg_ transfer after 21 d. **(A)** Representative images of the analyzed volume of interest of the healing bones are shown. +T_Reg_ treated mice clustered into responder (+T_Reg_ left, *n* = 4) and non-responder (+T_Reg_ right, *n* = 4). Control mice showed no complete bridging after 21 d (left, control, *n* = 6). In contrast, +T_Reg_ responder showed complete bridging, whereas non-responder showed no bridging. **(B–D)** Quantification of the μCT analysis, + T_Reg_ responder vs. + T_Reg_ non-responder vs. control mice. **(E–G)** Evaluation of the immune cell composition of +T_Reg_ responder vs. non-responder 21 d post-surgery. **(E)** Confirmation of CD4+ T_Reg_ engraftment in both, +T_Reg_ responder and non-responder compared to the control. **(F,G)** +T_Reg_ non-responder showed an elevated percentage of CD8+ T_EFF_ **(F)** as well as a significantly higher ratio of CD8+ T_EFF_ /CD4+ T_Reg_ **(G)** in the peripheral blood in comparison to the +T_Reg_ responder. TV, total volume; BV, bone volume; BV/TV, ratio of BV to TV; Mann–Whitney *U*-test, Bonferroni correction **(B–E)**.

Similar to the T_Reg_ transfer in SPF housed animals, we used mice with an experienced adaptive immunity to evaluate the therapeutic potential of CD4+ T_Reg_ to further bone regeneration. Animals received CD4+ T_Reg_ isolated from pooled donor mice to prevent heterogeneity of individual T_Reg_ donors (Figure 2A; +T_Reg_). In contrast to the clear data received from the SPF housed mice, non-SPF housed animals displayed a heterogeneous clustered healing outcome after CD4+ T_Reg_ transfer and osteotomy (Figures 2A–D). Remarkably, in half of the mice, referred to as +T_Reg_ responders, healing was significantly enhanced compared to control animals, illustrated by an increased TV (*p* = 0.01), BV (*p* = 0.01) and ratio of BV to TV (*p* = 0.038). In contrast, in the other half of the mice, the healing was not improved, but even showed a worsening (named “+T_Reg_ non-responders”) with significantly reduced TV (*p* = 0.01) and BV (*p* = 0.01).

In order to determine the reason behind the distinct healing differences of + T_Reg_ responders and non-responders several possibilities were analyzed. Differences in the functionality of the transferred T_Reg_ can be ruled out as we used pooled cells from several donor animals and saw responders and non-responders among the recipients of the same cell pool. Recipients were chosen from the same cage and randomly placed within the treatment groups—no correlation with responder or non-responder were found. Up to four animals were operated on at each surgery day and no correlation with the surgical sequence was found. Subsequently, we analyzed whether adoptively transferred T_Reg_ showed reduced engraftment in the non-responder mice. However, as shown in Figure 2E, the number of T_Reg_ detectable at day 21 post T_Reg_ infusion and osteotomy did not show a heterogeneous distribution and non-responder and responder mice showed comparably elevated T_Reg_ level.

Interestingly, upon analysis of the immune cell composition higher frequencies of CD62L-CD44+CD25+ activated CD8+ T_EFF_ cells were found in the non-responder animals, demonstrating that the immune experience within the group of mice even within the same cage was highly individual. A significantly elevated T_EFF_/T_Reg_ ratio in peripheral blood of +T_Reg_ non-responder vs. responder mice (*p* = 0.029; Figures 2F,G), indicating that the T_Reg_ treatment was not sufficient to reshape the negative effect of the CD8+T_EFF_ cells in the non-responder animals.

**Figure 3:**
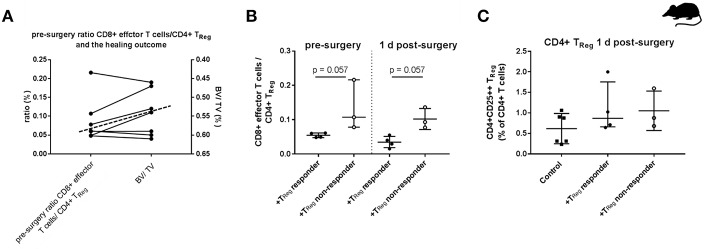
Individual effector/regulatory T cell ratio pre-osteotomy determines the healing outcome in mice. **(A)** Mice having a lower pre-surgery CD8+ T_EFF_ to CD4+ T_Reg_ ratio (left y-axis) showed a higher BV/TV (right y-axis, axis is reversed for a clearer depiction). Dashed line indicates the cut off between the +T_Reg_ responder (below the dashed line) and the +T_Reg_ non-responder (above the dashed line). **(B)** Adoptive CD4+ T_Reg_ transfer did not reshape the unfavorable CD8+ T_EFF_/CD4+ T_Reg_ ratio. **(C)** Engraftment of CD4+CD25++ T cells 1 d post-surgery is elevated in both, the +T_Reg_ responder (*n* = 4) and +T_Reg_ non-responder (*n* = 3) in comparison to the control group (*n* = 6). 45BV, total bone volume; TV, total callus volume; BV/TV, ratio of BV to TV; Mann–Whitney *U*-test.

To test this assumption, we analyzed this ratio of T_EFF_/T_Reg_ pre-operatively in the peripheral blood of another cohort of seven non-SPF housed mice. We performed the adoptive CD4+ T_Reg_ transfer, set the osteotomy and evaluated the healing outcome after 21 d as described before. Within this second cohort of osteotomized mice the heterogeneous healing outcome of the previous experiment was confirmed. The healing outcome 21 days after osteotomy again showed +T_Reg_ responder and +T_Reg_ non-responder. Four mice could be clearly categorized as +T_Reg_ responders (good healing outcome: bridging of the cortices in progress) and three mice were classified as +T_Reg_ non-responders (poor healing outcome: no bridging of the cortices). Based on this grouping, we compared values of the pre-surgery CD8+T_EFF_/CD4+T_Reg_ cell ratio with the μCT evaluation after 21 d (BV/TV) (Figure 3A). Notably, all four responder mice had a pre-surgery ratio of CD8+ T_EFF_/CD4+ T_Reg_ of <0.07 (below the dashed line) which consistently correlated with a higher BV/TV in comparison with the non-responder mice which showed a ratio of 0.08–0.23 (above the dashed line). We further evaluated this CD8+ T_EFF_/CD4+ T_Reg_ ratio at 1 d post-surgery. Remarkably, CD4+ T_Reg_ transfer could not reverse the unfavorable ratio in the non-responder mice (Figure 3B). Again, we could exclude unsuccessful engraftment of the adoptively transferred CD4+ T_Reg_ as shown in Figure 3C.

The presented data suggest that successful bone regeneration depends on the balance between CD8+ T_EFF_/CD4+ T_Reg_. The cut off between +T_Reg_ responder and +T_Reg_ non-responder however is not pronounced. In order to confirm the results from the mouse experiment that the CD8+ T_EFF_/CD4+ T_Reg_ ratio is a decisive factor for the bone healing outcome the immune cell composition in a patient cohort with a known healing outcome was analyzed.

### Elevated CD8+ T_EMRA_ Level in the Peripheral Blood of Patients With Impaired Healing After Bone Fracture

**Figure 4:**
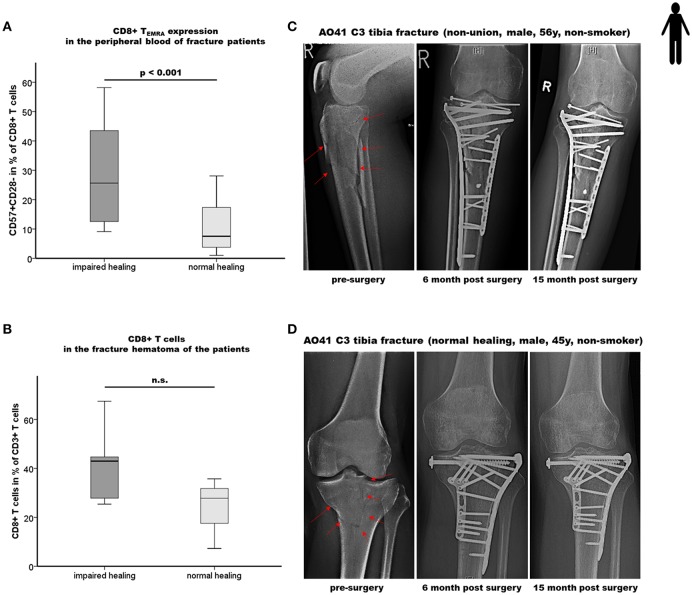
Impaired bone healing correlates with an elevated CD8+ T_EMRA_ level in both the peripheral blood as well as in the local fracture hematoma of patients. **(A)** Results of the flow cytometry analyses of terminally differentiated CD8+ effector T cells (CD8+T_EMRA_) in the peripheral blood of fracture patients at the time of operation are shown. Impaired fracture healing patients showed a significantly higher frequency of CD45+CD3+CD8+CD57+CD28- T cells (CD57+CD28-+ in % of CD45+CD3+CD8+ T cells) compared to the normal healing patients indicates a dramatic increase of CD8+ T_EMRA_: n = 12 for the impaired healing group, n = 23 for the normal healing group. **(B)** Quantification of CD8+ T cells in the hematoma region of fracture healing patients by epigenetic qPCR analysis. Impaired fracture healing patients showed an elevated level of CD8+ T cells in the local fracture area: n = 5 for the impaired healing group, n = 3 for the normal healing group **(C,D)** Representative x-ray images from the study cohort over a period of 15 months including the pre-surgery as well as the 6 and 15 months post-surgery states of fracture healing of an impaired healing **(C)** and a normal healing **(D)** patient, respectively. Student's t-test.

In accordance with the mouse experiment, a patient cohort with a high CD8+ T_EFF_ level should be included in the experiment to confirm our mouse data. Therefore, a new patient cohort was recruited applying our previously published (17) strategy to define delayed healing by functional and radiological methods (Figures 4C,D). In a first step elevated CD8+ T_EFF_ level were confirmed in the new patient cohort, the cells were further subtyped as T_EMRA_ cells CD45+CD3+CD8+CD57+CD28- (Figure 4A). The impaired healing group showed an almost three times higher proportion of circulating CD45+CD3+CD8+CD57+CD28- T_EMRA_ cells (*p* < 0.001).

### Enhanced Accumulation of CD8+ T Cells in the Fracture Hematoma of Patients With Impaired Fracture Healing

To further confirm the effect of the systemically found elevated T_EMRA_ cell count the fracture hematoma of these patient was evaluated for these cells. To address this issue, we applied a recently developed, easily applicable advanced technology to analyze immune cell subset composition from tissue samples based on CD3+-, T_Reg_- and CD8+-specific epigenetic pattern at tissue-derived DNA (30–32). Fracture hematoma composition does not simply reflect the repertoire of blood leukocytes but is enriched by T_EFF_ cells attracted actively to the fracture environment. The almost two times higher levels of CD8+ T cells in the hematoma of impaired vs. normal healing patients support the hypothesis that CD8+ T_EFF_ accumulate in the fracture hematoma, although statistical significance was not yet reached as only a low number of fracture hematoma samples was available for the analysis (Figure 4B).

**Figure 5:**
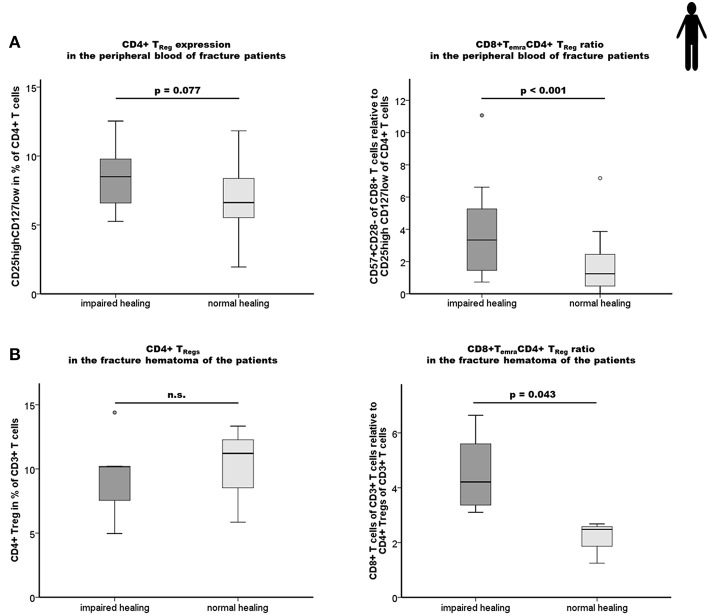
Impaired fracture healing correlates with an elevated level of CD4+ T_Reg_ and a higher ratio of CD8+ T_EMRA_/CD4+ T_Reg_. **(A)** Results of the flow cytometry analyses of the CD4+ T_Reg_ and the ratio CD8+T_EMRA_/CD4+T_Reg_ in the peripheral blood of fracture patients at the time of operation are shown. Impaired fracture healing patients showed slightly elevated levels of CD45+CD3+CD4+CD25highCD127low T_Reg_ (CD25highCD127low in % of CD45+CD3+CD4+) compared to the normal healing patients indicates a partial, but not sufficient compensatory effect. The ratio of CD8+T_EMRA_/CD4+T_Reg_ was significantly higher in patients with a delayed healing: *n* = 12 for the impaired healing group, *n* = 23 for the normal healing group. **(B)** Impaired fracture healing patients showed a significantly higher ratio of CD8+T cells/CD4+ T_Reg_ in the hematoma region demonstrating an insufficient compensatory effect of the CD4+ T_Reg_ in the impaired fracture healing patients: *n* = 5 for the impaired healing group, *n* = 3 for the normal healing group. Student's *t*-test.

In a next step, we analyzed the observed tight interconnectivity between the CD8+ T_EFF_ and CD4+ T_Reg_ ratio and the bone healing outcome in our human patient cohort. Interestingly, we observed an elevated percentage of CD4+ T_Reg_ in the peripheral blood of impaired healing patients. This could be interpreted as an intrinsic effort to counteract the higher CD8+ T_EFF_ level that was however, not able to reverse the negative impact of the high CD8+ T_EMRA_/CD4+ T_Reg_ ratio in those patients (Figure 5A; *p* < 0.001). This result is in accordance with the data of the mouse experiment. Remarkably, this imbalance of CD8+ T_EFF_/CD4+ T_Reg_ was also reflected in the facture hematoma (Figure 5B). Impaired fracture healing patients showed a significantly higher CD8+ T cells/CD4+ T_Reg_ ratio without overlap between the two groups, ranging from 3.0 to 6.0 and 1.2 to 2.7 in patients who showed an impaired and normal healing outcome, respectively (*p* = 0.043).

These findings demonstrate that the ratios of CD8+ T_EFF_ to CD4+ T_Reg_ in the peripheral blood are mirrored by those within the fracture hematoma. This cellular correlation between the periphery and the site of injury further highlights the significance of the balance of pro-inflammatory CD8+ T_EFF_ to anti-inflammatory CD4+ T_Reg_ for a successful bone healing process.

## Discussion

Our study demonstrates the relevance of the T cell subset composition for regeneration using bone healing in a murine osteotomy model as well as in patients. Enhanced CD8+ T_EFF_/CD4+ T_Reg_ ratios both in peripheral blood and locally in the fracture hematoma were associated with an impaired bone healing outcome.

The data indicates that the T_EFF_/T_Reg_ ratio could be a predictive biomarker for bone healing. However, as flow cytometry analysis from hematoma is hardly applicable for the clinical routine, we applied a recently developed fast epigenetic method allowing T cell subset quantification in tissue specimen (30–32) that could be further developed into an intraoperative diagnostic tool. This would enable a stratification of patients in potential need of additional treatments at the time of the initial fracture treatment.

This study clearly demonstrated that the model used for an experimental evaluation of a hypothesis should be carefully considered. While the results in the SPF housed mice indicated that a treatment enhancing T_Reg_ percentages shows 100% healing enhancement changing the model to one with a higher immune experience (and thus a model that is closer to the patient situation) changed this result to only 50% healing success. What is even more alarming is that the other 50% instead of showing an unchanged healing outcome revealed a significantly impaired healing. This indicates that in cases of an unbalanced immune response otherwise supportive immune cells can turn into detrimental cells for the healing process.

As in human beings, non-SPF housed mice, even if kept in the same cage, show an intragroup heterogeneity regarding the T_EFF_/T_Reg_ balance in the blood and secondary immune organs. In mice with very high T_EFF_/T_Reg_ ratio the adoptive transfer of T_Reg_ could not rescue this imbalance measured immediately after cell transfer in blood samples as well as at the end of 21 d follow-up in immune organs even though engraftment was confirmed. This unsuccessful rebalancing of the T_EFF_/T_Reg_ ratio was associated not only with a lack of benefit of the T_Reg_ transfer but worsened the healing even more. For our immune-experienced mice, we applied a relative short exposure time of 4 weeks in the non-SPF housing. Therefore, the observed findings and differences in the non-SPF mice in comparison to the SPF mice are even more astonishing. In comparison, the changes in the adaptive immune system visible after 4 weeks of exposure were not as pronounced as seen in an aged human being. Therefore, the ratios in the mouse model cannot be transferred toward patients.

How can T_EFF_ cells affect bone healing? In fact, we could recently show (17) that CD8+ T_EFF_ produce high amounts of inflammatory cytokines, such as IFNγ and TNFα even without costimulatory signals. Their strong inflamed tissue homing properties allow them to deliver those cytokines at fracture sites. Although some local inflammation is beneficial for triggering endogenous regeneration post-fracture (38), too much of “a beautiful thing” can have worsening effects, as shown by inhibition of differentiation of osteogenic precursors in the presence of supernatants of CD8+ T_EFF_, an effect that could be converted by neutralizing TNFα and IFNγ (17).

How might T_Reg_ control the T_EFF_ response in at least half of the immune-experienced mice with pre-established T_EFF_ cells? The positive effects of T_Reg_ might be explained by the prevention of an imbalanced T_EFF_/T_Reg_ ratio as a result of T_Reg_ blocking memory/effector T cells at multiple checkpoints (34): T_Reg_ can inhibit the cytokine release and proliferation of T_EFF_ cells by cell-cell contact dependent and independent mechanisms. This seems to be related at least partly to adenosine formation and metabolic competition (e.g., tryptophan pathway) as shown before (34).

**Figure 6:**
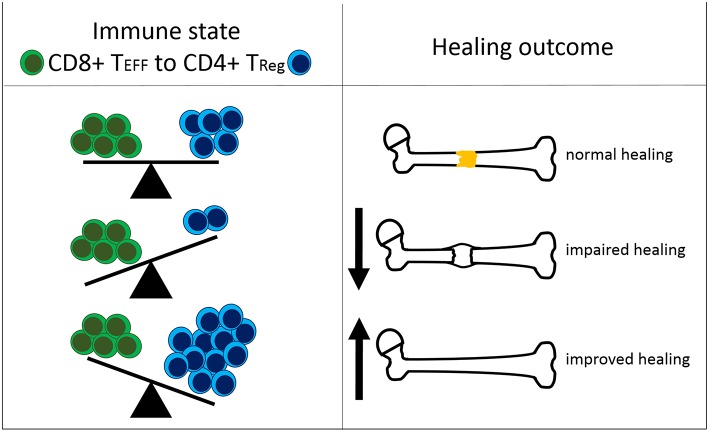
Interdependency of the immune status and the healing outcome after bone fracture. The ratio of CD8+ effector T cells (CD8+ T_EFF_) to CD4+ regulatory T cells (CD4+ T_Reg_) affects the healing outcome after bone fracture. Under normal healing conditions, the T cell ratio is balanced. An elevated amount of CD8+ T_EFF_ relative to CD4+ T_Reg_ is unfavorable for successful healing. A shifted balance in the CD4+ *T*_*Reg*_*sb* direction improves bone fracture healing.

In summary, our findings confirm our recent data that suggested a negative impact of CD8+ T_EMRA_ on bone fracture healing in patients (17) but extend these observations further by demonstrating the relevance of the systemic and local balance between effector and regulatory mechanisms (T_EFF_/T_Reg_ ratio). Moreover, our data indicate that a CD4+ T_Reg_ based immunomodulation is feasible to further bone regeneration but its efficiency is dependent on the recipient's immune status, especially of a balanced adaptive immunity (Figure 6).

The strong link between the pre-surgery immune cell ratio and the healing outcome was clearly demonstrated in our animal model.

Considering the fact that the non-responder mice show the tendency of even worse healing compared to the responder and even compared to the control mice the following considerations could be made. It is already well-known that CD4+ T_Reg_ are not a committed stable T cell subpopulation. Dependent on the inflammatory environment, several studies reported a loss of FOXP3 expression by CD4+ T_Reg_ accompanied by the loss of their suppressive activity (39, 40). Furthermore, CD4+ T_Reg_ cannot only lose their suppressive capacity but can also convert to CD4+ T_EFF_ cells such as Th17 cells (40–42). For Th17 cells controversial effects on bone cells have been reported. They can stimulate the formation of osteoclasts by the production of Receptor Activator of NF-κB Ligand (RANKL), either directly by themselves or indirectly via osteoblasts or synovial fibroblasts. Furthermore, it is described that Th17 cells act pro-osteogenic. Additionally, in the context of several bone related disorders, the underlying cause is postulated to be an imbalance of the ratio between Th17 cells and CD4+ T_Reg_ (43, 44).

*In vitro* studies using murine or human CD4+ T_Reg_ have demonstrated a possible transition between CD4+ T_Reg_ and IL-17 producing (Th17) T cells. For example, Xu et al. reported that this transition is IL-6 dependent (41). Using co-culture setups, another study showed that murine CD4+ T_Reg_ can obtain the expression of Rorγt, the master transcription factor of Th17 cells (45). Studies with human CD4+ T_Reg_ also revealed this conversion between CD4+ T_Reg_ and Th17 cells (46, 47). One possible explanation for the observed non-responsiveness of some CD4+ T_Reg_ enriched animals could, therefore, be a potential transition of the pro-regenerative CD4+ T_Reg_ into an anti-regenerative Th17-like phenotype. This hypothesis could be more directly verified by findings from Zhou and colleagues (*40*). They evaluated the stability and fate of (initially) Foxp3+ CD4+ T_Reg_ under homeostatic as well as pathogenic conditions *in vivo* by using a combined Foxp3-GFP-Cre/ R26-YFP mice system. This mouse model enables the detection of the induction or downregulation of Foxp3 expression and further allows to track the cell fate of Foxp3+ CD4+ T_Reg_. Zhou and colleagues showed that the strength of loss of Foxp3 expression from CD4+ T_Reg_ was dependent on the microenvironment and was stronger in inflamed tissues in autoimmune conditions. Furthermore, they showed that these converted Foxp3- “CD4+ T_Reg_” express memory cell marker and pro-inflammatory cytokines like IFNγ and IL-17. Thus, there is evidence that a strong pro-inflammatory microenvironment bears the risk to promote the transition of Foxp3+ to Foxp3- CD4+ T cells. If such a transition is happening during bone fracture healing in elevated pro-inflammatory conditions, this could explain the observed impaired healing in our mice where CD4+ T_Reg_ transfer did not overcome the pro-inflammatory microenvironment resulting from the high T_EFF_ levels.

## Conclusion

Bone fracture healing is a highly complex process. The presented and discussed human and mice data demonstrate the strong interdependency between the adaptive immunity and the bone system in the context of bone healing. They further highlight how well-regulated the interplay of different (immune) cell subtypes has to be to promote regeneration. Due to the observed findings at both sides, locally at the fracture as well as systemically in the peripheral blood, this cellular interplay is probably not only crucial for bone fracture repair but also for the healing capacity of other injured tissues. With regard to clinical translation, our data indicate the advantage of a T_EFF_/T_Reg_ ratio analysis, allowing to identify patients at risk in an early stratification and thereby predict already preoperatively the healing potential after bony injury. A prospective multicenter study (>600 patients) is currently ongoing to confirm the promising potential of the CD8+ T_EMRA_/CD4+ T_Reg_ ratio as a potential biomarker for predicting the healing outcome in human bone fracture patients. Therefore, the cellular interplay could be used to better understand regulatory mechanisms guiding regenerative processes and thus could reveal possible novel target points for (immunomodulatory) treatment strategies to therapeutically support and improve impaired tissue repair.

## Data Availability

All datasets generated for this study are included in the manuscript/Supplementary Files.

## Ethics Statement

The study was performed in compliance with the International Conference on Harmonization Guidelines for Good Clinical Practice and the Declaration of Helsinki. All patients participated with a written informed consent, and the study was approved by the Institutional Review Board (IRB) of the Charité – Universitätsmedizin Berlin (IRB approval EA2/096/11). All animal experiments were approved by the local legal representatives (Landesamt für Gesundheit und Soziales Berlin: G0008/12; T0119/14; T0249/11) and done accordingly to the guidelines of the Animal Welfare Act, the National Institutes of Health Guide for Care and Use of Laboratory Animals, and the National Animal Welfare and ARRIVE Guidelines.

## Author Contributions

CS, KS-B, SR, SG, GD, and H-DV: conceptual idea and design of the study and drafting manuscript. CS and SR: data collection, analysis, and interpretation. CS and KS-B: clinically relevant osteotomy model. CG: hematoma preparation. BS, UB, and CG: epigenomic DNA data collection and analysis. SM, MD, and CK: clinical evaluation of patients and sample harvesting. CB: flow cytometry. All authors revised the final version of the manuscript.

### Conflict of Interest Statement

BS and UB were employed by the company Epiontis GmbH, Precision for Medicine Group, Berlin, Germany. The remaining authors declare that the research was conducted in the absence of any commercial or financial relationships that could be construed as a potential conflict of interest.
