# Supplementary material for: Individual Effector/Regulator T Cell Ratios Impact Bone Regeneration
Source: Front Immunol. 2019 Aug 16;10:1954. doi: 10.3389/fimmu.2019.01954 (PMC6706871; doi:10.3389/fimmu.2019.01954)
Supplement: Supplementary file 1 [file Data_Sheet_1.docx]

Supplementary Materials:

**Figure S1:** Study design of the mouse experiment


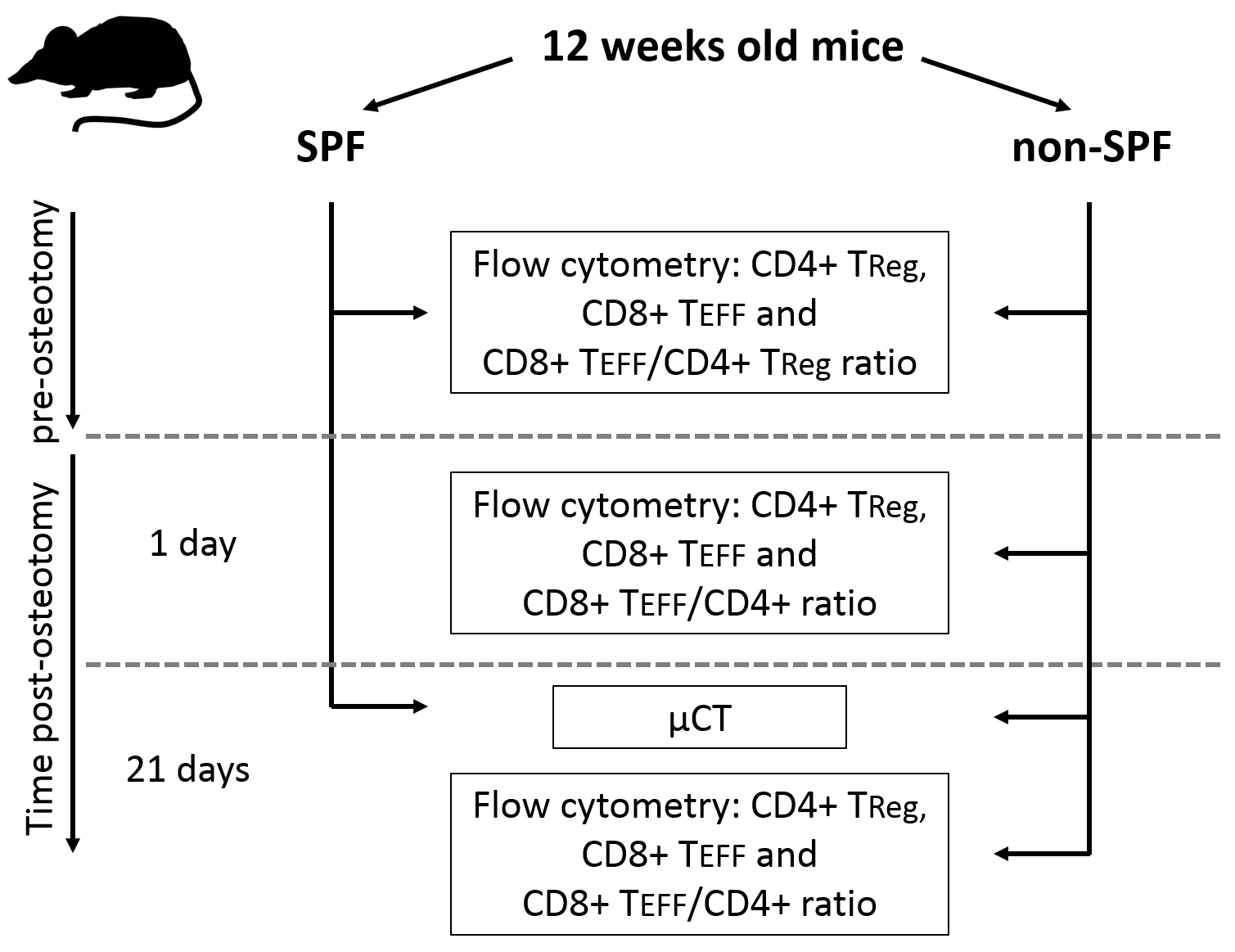


Supplemental Figure 1: Scheme of the experimental design of the mice experiments

**Figure S2:** Gating strategy used in the flow cytometry analysis

A: Gating strategy for the CD8 T cell subsets:


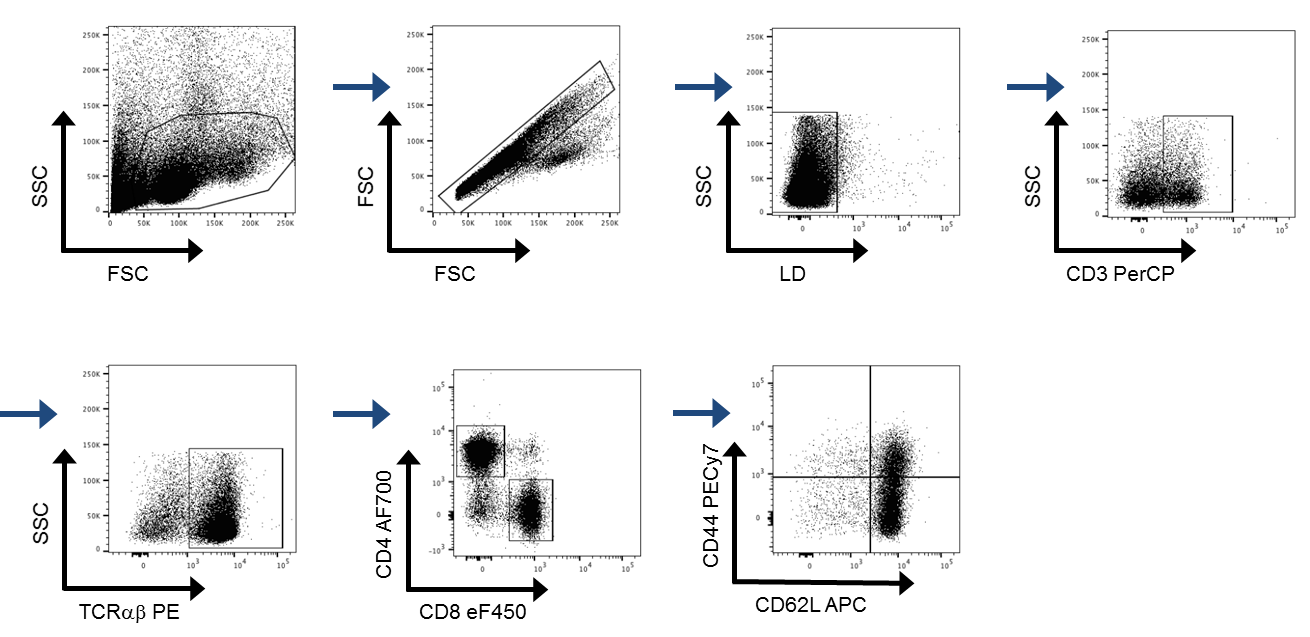


B: Gating for the regulatory T cell subset


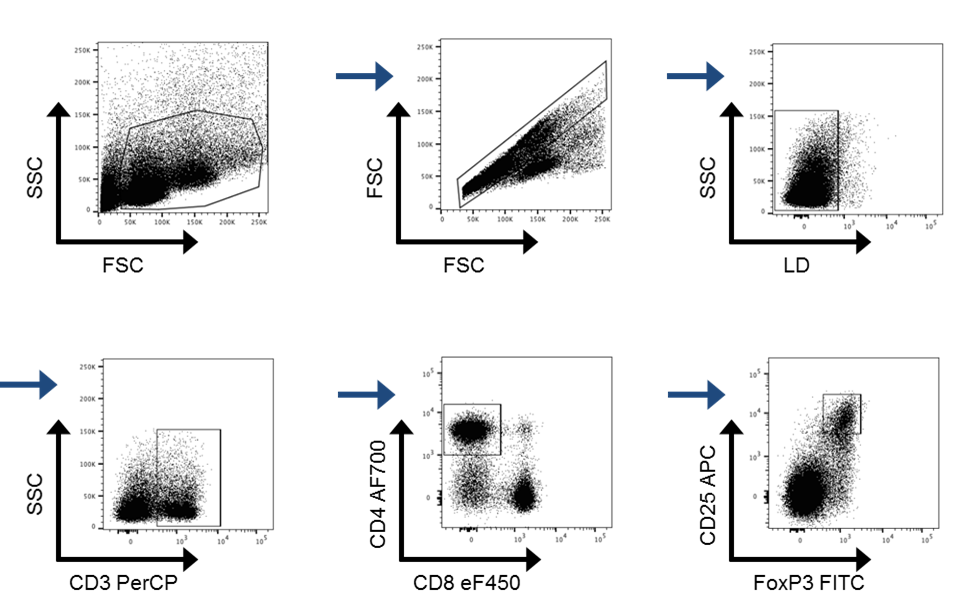


**Figure S3:** Purity of CD4+ TReg after MACS isolation


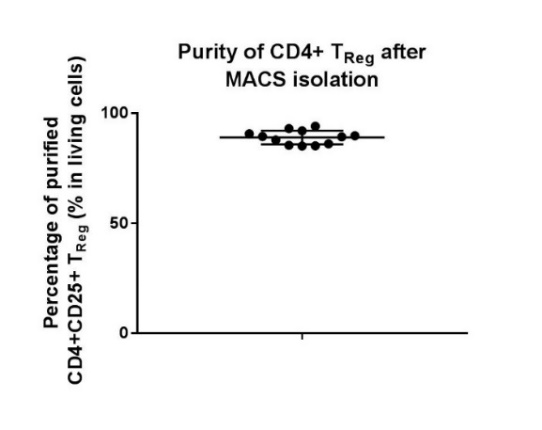


Supplemental Figure 3: Purity of CD4+ TReg after MACS isolation. MACS isolated CD4+ TReg were analyzed via flow cytometry for the expression of FoxP3 to confirm the CD4+ TReg phenotype. Displayed is the percentage in living cells. n = 12

**Figure S4:** Immune cell composition in SPF vs. non-SPF mice


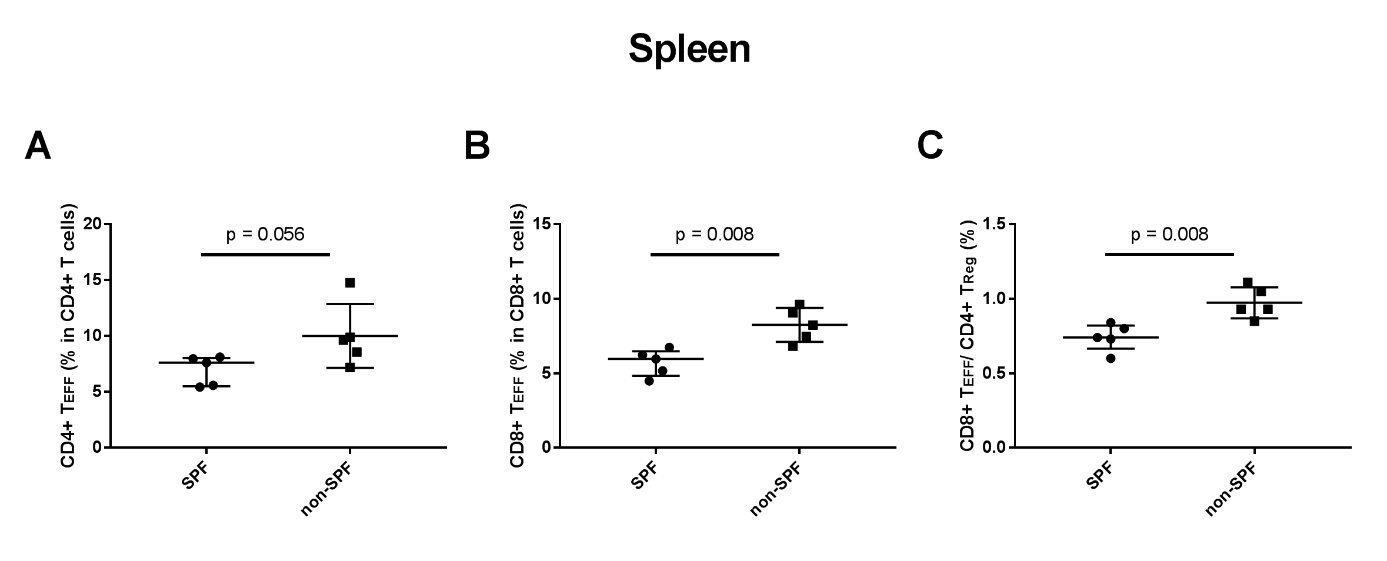


Supplemental Figure 4: Flow cytometry analyses of the immune cell composition in the spleen of SPF (n = 6) and non-SPF housed mice (n = 6). Non-SPF mice displayed a higher percentage of CD4+ TEFF (A), CD8+ TEFF (B) as well as a higher ratio of CD8+ TEFF/CD4+ TReg in comparison to SPF housed mice. Mann-Whitney U test

**Table S1: Overview of the fracture types of each individual patient**

| **Nr.** | **Fx-Type** | **Fx location** | **Age** | **Sex** | **group** |
| --- | --- | --- | --- | --- | --- |
| 1 | AO41 C2 | Tibia | 69 | female | Impaired |
| 2 | AO41 B3 | Tibia | 61 | male | Impaired |
| 3 | AO43 B2 | Tibia | 47 | female | Impaired |
| 4 | AO41 C3 | Tibia | 26 | male | Impaired |
| 5 | AO41 C3 | Tibia | 56 | male | Impaired |
| 6 | AO41 C3 | Tibia | 74 | female | Impaired |
| 7 | AO41 C3 | Tibia | 50 | female | Impaired |
| 8 | AO11 C2 | Humerus | 49 | male | Impaired |
| 9 | AO12 B1 | Humerus | 68 | male | Impaired |
| 10 | AO11 A3 | Humerus | 47 | male | Impaired |
| 11 | AO13 C3 | Humerus | 55 | female | Impaired |
| 12 | AO 62 C2 | Acetabulum | 53 | male | Impaired |
| 13 | AO41 B2 | Tibia | 35 | female | normal |
| 14 | AO41 B3 | Tibia | 60 | male | normal |
| 15 | AO41 C1 | Tibia | 53 | female | normal |
| 16 | AO41 B3 | Tibia | 71 | male | normal |
| 17 | AO41 B3 | Tibia | 43 | male | normal |
| 18 | AO41 C3 | Tibia | 59 | female | normal |
| 19 | AO41 B2 | Tibia | 45 | female | normal |
| 20 | AO41 C3 | Tibia | 57 | male | normal |
| 21 | AO41 C3 | Tibia | 19 | male | normal |
| 22 | AO41 B3 | Tibia | 51 | male | normal |
| 23 | AO41 C1 | Tibia | 52 | female | normal |
| 24 | AO41 B3 | Tibia | 43 | female | normal |
| 25 | AO41 B1 | Tibia | 61 | male | normal |
| 26 | AO41 B3 | Tibia | 53 | male | normal |
| 27 | AO41 C3 | Tibia | 75 | female | normal |
| 28 | AO41 C3 | Tibia | 45 | male | normal |
| 29 | AO41 B3 | Tibia | 44 | male | normal |
| 30 | AO41 B3 | Tibia | 49 | male | normal |
| 31 | AO11 B3 | Humerus | 46 | female | normal |
| 32 | AO11 B1 | Humerus | 64 | female | normal |
| 33 | AO11 C1 | Humerus | 63 | female | normal |
| 34 | AO11 C1 | Humerus | 45 | female | normal |
| 35 | AO62 C2 | Acetabulum | 77 | male | normal |

**Table S2: Characteristics of fracture treatment in patients**

| **Parameter** | **Impaired Healing Patients (n=12)** | **Normal Healing Patients**  **(n= 23)** | **p -value** |
| --- | --- | --- | --- |
| Primary Injury (closed) | 100% (12/12) | 100% (23/23) | 1.0 |
| Fracture Type AO - A | 8.3% (1/12) | 0% (0/23) | 0.1 |
| Fracture Type AO - B | 25% (3/12) | 56.5% (13/23) |  |
| Fracture Type AO - C | 66.6% (8/12) | 45.5% (10/23) |  |
| ASA class one or two | 75.0% (9/12) | 87% (20/23) | 0.373 |
| ASA class three | 25.0% (3/12) | 13% (3/23) |  |
| Number of interventions (0-1) | 33.3% (4/12) | 87% (20/23) | **0.003** |
| Number of interventions (2-4) | 50.0% (6/12) | 13% (3/23) |  |
| Number of interventions  (> 4) | 16.6.% (2/12) | 0% (0/23) |  |
| Surgery method (percutaneous) | 0% (0/12) | 0% (0/23) | 1.0 |
| Surgery method (Pin) | 0% (0/12) | 0% (0/23) |  |
| Surgery method (ORIF) | 100% (12/12) | 100% (23/23) |  |
| Soft tissue defect  (uneventful surgery) | 33.3% (4/12) | 39.1% (9/23) | 0.466 |
| Soft tissue defect  (small treatment) | 50.0% (6/12) | 56.5.% (13/23) |  |
| Soft tissue defect  (complex treatment) | 16.7% (2/12) | 4.3% (1/23) |  |
| Soft tissue defect  (poor vascularity) | 0% (0/12) | 0% (0/23) |  |
| Clinical infection status  (without post-operative infection) | 91.7% (11/12) | 91.3% (21/23) | 0.971 |
| Clinical infection status  (post-operative infection) | 8.3% (1/12) | 8.7% (2/23) |  |
| Steroid/NSAID Therapy | 66.6% (8/12) | 73.9% (17/23) | 0.652 |
